# MicroRNA-152 suppresses cisplatin resistance in A549 cells

**DOI:** 10.3892/ol.2020.11422

**Published:** 2020-02-28

**Authors:** Wenfei Zhao, Hongyun Li, Shanshan Yang, Di Guo, Jing Chen, Shaoyi Miao, Yi Xin, Miaomiao Liang

Oncol Lett 18: 4613-4620, 2019; DOI: 10.3892/ol.2019.10834

Following the publication of this article, the authors have realized that they erroneously overlooked including details of the funding that they received for their research in the Declarations section of their paper. In this section, the authors should have indicated that their research was funded by the Key Scientific and Technology Project of Henan Province (grant no. 162102310133).

The authors apologize to the funders of their research project, and to the readership of the Journal for any inconvenience caused.

